# Real-world incidence of severe infections in multiple myeloma patients receiving bispecific antibodies: a meta-analysis

**DOI:** 10.1007/s00277-026-07026-9

**Published:** 2026-04-27

**Authors:** Federico Spataro, Vanessa Desantis, Hermann Einsele, Franco Dammacco, Angelo Vacca, Roberto Ria, Antonio Giovanni Solimando

**Affiliations:** 1 Department of Precision and Regenerative Medicine and Ionian Area - DiMePRe-J, Section of Pharmacology, University of Bari Aldo Moro, Bari, 70124 Italy; 2 Department of Internal Medicine II, University Hospital, Wurzburg, Germany; 3 Department of Precision and Regenerative Medicine and Ionian Area - DiMePRe-J, Guido Baccelli Unit of Internal Medicine, School of Medicine, University of Bari Aldo Moro, Bari, 70124 Guido Italy

**Keywords:** Multiple myeloma, Bispecific, BiTEs, Infection

## Abstract

Bispecific antibodies (BiTEs) have transformed the therapeutic landscape of relapsed/refractory multiple myeloma (RRMM), offering deep and durable responses even in heavily pretreated patients. However, their use carries substantial infectious risk due to immunosuppression, particularly hypogammaglobulinemia, induced by T-cell-redirecting therapy. To define the real-world incidence of grade 3–4 infections, we conducted a systematic review and meta-analysis of retrospective studies including RRMM patients treated with BiTEs currently approved for clinical use. Ten studies encompassing 1,373 patients were analysed using a random-effects model of pooled proportions. The overall rate of grade 3–4 infections was 0.25 (95% CI, 0.22–0.30) after a median follow-up of 8.3 months, with moderate heterogeneity (I²=52.9%). Subgroup analyses showed comparable pooled event rates for teclistamab—anti-CD3/BCMA—(0.26; 95% CI, 0.22–0.31) and talquetamab—anti-CD3/GPRC5D—(0.23; 95% CI, 0.14–0.33). Meta-regression revealed an inverse association between infection rates and mean number of prior therapy lines (*p* = 0.002) and prior BCMA exposure (*p* = 0.0019), likely because, in real world setting, clinicians select fitter, immunologically stable patients for BiTEs therapy. In summary, approximately one in four RRMM patients receiving BiTEs experiences severe infection. These findings underscore the need for proactive monitoring and standardized preventive measures, including immunoglobulin replacement, prophylaxis, vaccination, to ensure safe, effective integration of BiTEs into routine MM care.

**Supplementary Information:**

The online version contains supplementary material available at 10.1007/s00277-026-07026-9.

## Introduction

Multiple myeloma (MM) is a malignant plasma cell disorder characterized by clonal proliferation within the bone marrow, leading to end-organ damage and profound immune dysfunction [1]. Infections are a leading cause of morbidity and mortality from diagnosis throughout the disease course, due to both disease-related immune impairment and treatment-induced immunosuppression [2]. Although proteasome inhibitors, immunomodulatory drugs, and monoclonal antibodies, MM remains incurable, and most patients eventually relapse. For heavily pretreated individuals, bispecific antibodies (BiTEs) are a promising immunotherapy, redirecting cytotoxic T cells against malignant plasma cells via dual antigen targeting [3].

Currently, several BiTEs are approved for clinical use in relapsed/refractory MM (RRMM): teclistamab, elranatamab and the more recently approved linvoseltamab, all targeting B-cell maturation antigen (BCMA), and talquetamab - which targets the G protein-coupled receptor family C group 5 member D (GPRC5D). In pivotal trials, these agents achieved deep and durable responses in patients with limited options. In the phase 1/2 MajesTEC-1 study, teclistamab produced response rates > 60%, with grade 3–4 infections in up to 44.8% at 14 months’ median follow-up [4]. In the phase 2 MagnetisMM-3 trial, elranatamab showed comparable efficacy, with 39.8% grade 3–4 infections at 14.7 months [5]. In MonumenTAL-1, talquetamab was associated with infections in 56–76% of patients, and grade 3–4 events in 18–26%, depending on regimen and prior T-cell-redirecting therapy [6]. Real-world evidence remains heterogeneous across these agents and is currently most mature for teclistamab and talquetamab, for which larger retrospective cohorts and longer clinical experience are available.

BiTEs demonstrate strong efficacy but are associated with a substantial infectious risk, particularly in the context of disease-related immune dysfunction and treatment-induced immunosuppression [7]. Variability in infection rates and follow-up across studies limits direct comparisons and may underestimate the true burden.

Despite their growing clinical use, a comprehensive quantitative synthesis of real-world grade 3–4 infection rates with BiTEs is lacking. Available retrospective studies report heterogeneous findings in small cohorts. Therefore, this meta-analysis was focused on teclistamab and talquetamab, as they represent the only BiTEs for which the quantity and quality of available real-world evidence allow a robust quantitative synthesis.

## Methods

### Search strategy and selection criteria

**Fig. 1:**
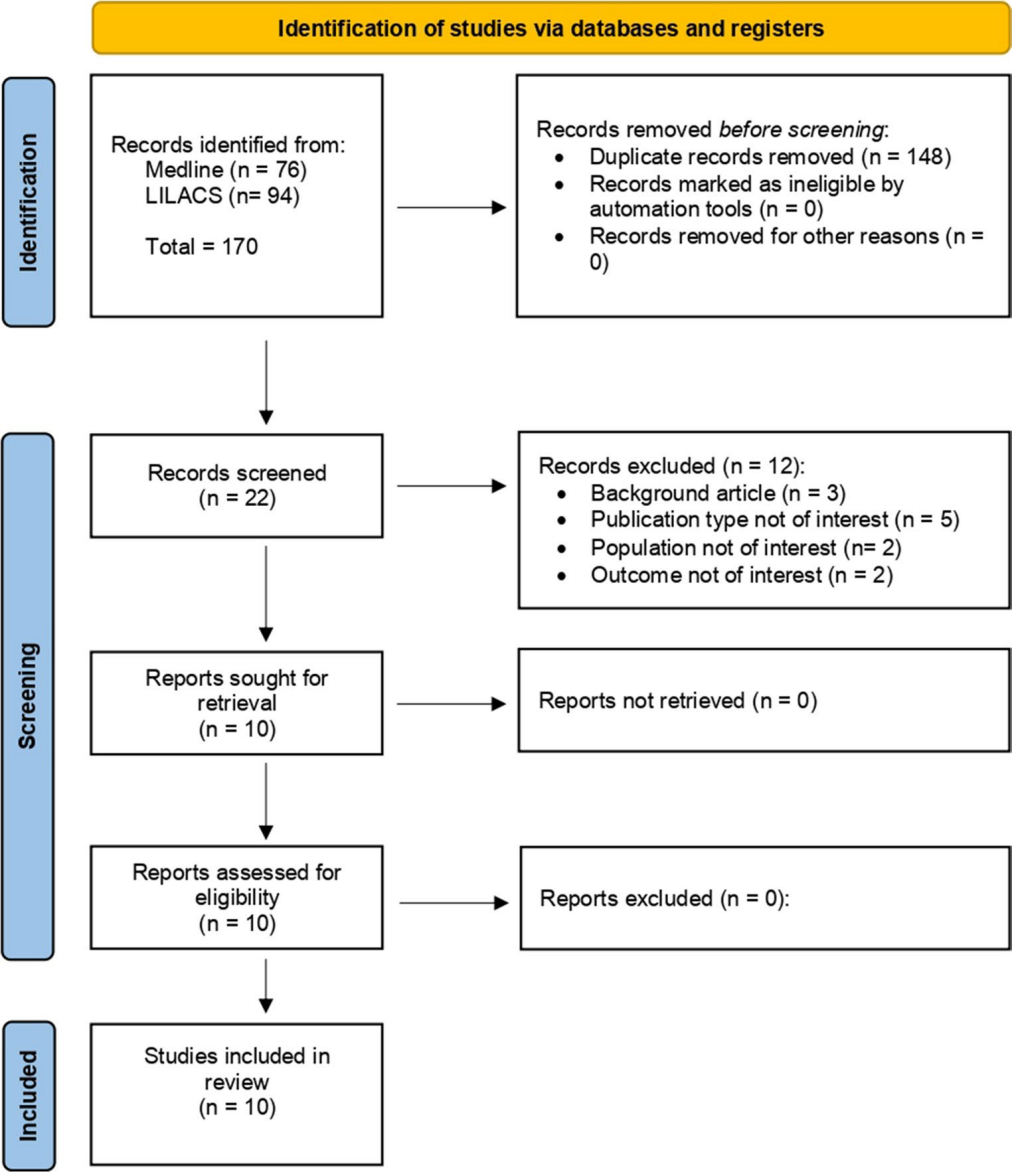
Flow diagram of research screening. Medline: ((((multiple myeloma[Title/Abstract]) OR (myeloma[Title/Abstract])) AND (((((bispecific[Title/Abstract]) OR (bites[Title/Abstract])) OR (teclistamab[Title/Abstract])) OR (elranatamab[Title/Abstract])) OR (talquetamab[Title/Abstract])) AND ((infection*[Text Word]) OR (sepsis[Text Word]))) NOT (review[Publication Type])) NOT (lymphoma[Title]). #1 Multiple myeloma [Ti/Ab] OR myeloma [Ti/Ab] - #2 bispecific [Ti/Ab] OR bites [Ti/Ab] OR teclistamab [Ti/Ab] OR elranatamab [Ti/Ab] OR talquetamab [Ti/Ab] - #3 infection [TW] OR sepsis [TW] - #4 review [PT] - #5 lymphoma [Ti]. #1 AND #2 AND #3 NOT #4 NOT #5. LILACS: ((“multiple myeloma” OR myeloma) AND (bites OR “bispecific”)) AND (“infection”) NOT review

This systematic review and meta-analysis was conducted according to the Preferred Reporting Items for Systematic Reviews and Meta-Analyses (PRISMA) guidelines [8]. The study protocol is registered in PROSPERO (registration ID: CRD420251231632). We searched MEDLINE and LILACS databases from inception (no backwards time limit) to October 1st, 2025, to identify studies evaluating the rate of severe infections in MM patients treated with BiTEs. The complete list of search terms is detailed in Fig. 1.

Studies were included if they met the following criteria: (1) retrospective design; (2) enrolled adult patients with MM receiving BiTEs therapy; (3) clearly specified the individual BiTEs agent administered (e.g., teclistamab or talquetamab); and (4) reported the number of patients who developed grade 3–4 infections. Studies lacking sufficient data for outcome extraction were excluded.

No restrictions were applied regarding publication date. In addition, the reference lists of included studies were manually screened to identify further relevant reports. Currently, most available real-world evidence on BiTEs therapy in MM derives from retrospective analyses; therefore, limiting inclusion to this study design minimized heterogeneity related to data collection methods and outcome assessment.

### Data collection process

Titles and abstracts were initially screened, followed by full-text review, data extraction, and independent assessment of study quality and risk of bias by two reviewers (FS and AGS) using the web-based platform Rayyan [9]. Discrepancies were resolved by consensus. For each eligible study, data were collected on study design and characteristics, clinical setting, inclusion criteria, patient population, intervention type, and reported outcomes.

### Outcomes

The primary outcome of interest was the proportion of patients who developed grade 3–4 infections during treatment with BiTEs [10]. Given that all included studies reported the number of infection events relative to the total number of treated patients, data were synthesized as pooled proportions (event rates) rather than comparative effect measures.

### Data analysis and risk of bias assessment

All statistical analyses were conducted using R software (version 4.3.1) [11]. A two-sided p-value < 0.05 was considered indicative of statistical significance. Between-study heterogeneity (*I*^2^) was assessed using the *I*² statistic [12]. A random-effects meta-analysis model was applied to estimate the pooled event rate and its 95% confidence interval (CI).

The summary of findings tables was created through the GRADEpro GDT software (available at *gradepro.org*). To assess the study quality, we applied the Quality Appraisal of Case Series Studies Checklist developed by the Institute of Health Economics (IHE) (accessible at http://www.ihe.ca/research-programs/rmd/cssqac/cssqac-about)*.* Responses were categorized as “yes,” “unclear/partial,” or “no.” Studies were deemed of acceptable quality (low to moderate risk of bias) if ≥ 70% of responses were “yes” [13].

Publication bias was evaluated through funnel plots visual inspection [14]. The quality of evidence was assessed using the GRADE approach [15].

Meta-regression analyses were conducted to explore potential sources of heterogeneity and to examine whether study-level characteristics influenced the proportion of severe (grade 3–4) infections. The following moderators were evaluated: median treatment duration (months), patient age, sex distribution, previous autologous stem cell transplantation (ASCT), percentage of extramedullary disease (EMD), high cytogenetic risk, International Staging System (ISS) stage I and III, prior exposure to BCMA-directed therapy, penta-refractory status, and use of immunoglobulin supplementation.

Robustness was assessed using leave-one-out analysis. Between-study heterogeneity was tested using the 𝝌² test and reported according to the *I²* statistic [16].

## Results

### Study selection

The bibliographic search yielded 170 records. After the initial screening and triage process, 10 articles met the inclusion criteria and were included in the meta-analysis (Fig. 1).

### Quality assessment and risk of bias

The overall quality for all outcomes was deemed acceptable (low risk of bias) in most studies. All 10 studies (100%) reported ≥ 70% “yes” responses according to the critical appraisal tool adopted (S-Table 1). The overall certainty of the evidence for the severe infection rate outcome was judged to be low (S-Table 2).

### Studies’ and patients’ characteristics

Table 1 summarizes the 10 studies included in the analysis. All studies had a retrospective design, and 9 out of 10 were multicenter. Three studies reported data stratified by subgroups. Specifically, Mian et al. [17] classified patients as fit (“Mian fit”) or frail (“Mian frail”); Stork et al. [18] divided patients into those receiving teclistamab every other week (“Stork nwd”) and those on a weekly dosing schedule (“Stork wd”); and Hammons et al. [19] distinguished between patients treated with talquetamab in combination with daratumumab and/or pomalidomide (“Hammons comb”) and those receiving talquetamab monotherapy (“Hammons mono”). For the study by Cani et al. [20], only the cohort of patients treated with talquetamab was included in the analysis, as the group described as receiving BCMA-targeted therapy did not clearly specify the individual agent administered.

**Table 1:** Studies’ characteristics at baseline

Study, year	Study type	Treatment	Patients at baseline, *n*°	Treatment duration, months (median)
Mohan, 2024	R, MC	teclistamab	110	3.5
Riedhammer, 2024	R, MC	teclistamab	123	6
Mian fit, 2025	R, MC	teclistamab	22	13.1
Mian frail, 2025			59	
Razzo, 2025	R, MC	teclistamab	509	10.1
Stork nwd, 2025	R, MC	teclistamab	18	5.9
Stork wd, 2025			55	4.7
Tan, 2025	R, MC	teclistamab	210	5.3
Yi, 2025	R, MC	teclistamab	42	16.4
Hammons comb, 2023	R, MC	talquetamab	15	5.6
Hammons mono, 2023			15	7.4
Frenking, 2024	R, MC	talquetamab	138	7.8
Cani, 2025	R, SC	talquetamab	57	4.5
Total			**1**,**373**	**8.3** ^*^

*Comb*, combination therapy; *MC*, multicenter; *mono*, monotherapy; *ndw*, non-weekly dosing; *SC*, single center; *wd*, weekly dosing

^*^Value represents the weighted mean of the medians reported in individual studies, weighted by the number of patients in each trial

^§^The denominator does not include patients that were not assessed for that clinical manifestation

Seven studies reported data on patients treated with teclistamab, while the remaining three focused on talquetamab [17–26].

The baseline patient population included 1,373 individuals. The sample size of the studies varied, ranging from 15 to 509 patients. Median treatment duration ranged from 3.5 to 16.4 months, with a weighted median duration of 8.3 months (Table 1). Females were 584 (42.9%), with a mean age of 67.9 years (Table 2). The overall mean number of prior lines of therapy across studies was 6.1. The pooled proportion of patients with prior autologous stem cell transplantation was 82%, while 34% presented with extramedullary disease. The prevalence of high-risk cytogenetic abnormalities was 48%, and 29.7% of patients were classified as ISS stage III (36.3% of patients were ISS stage I). Prior exposure to BCMA-directed therapy was observed in 51.7% of patients, and 51.7% were penta-refractory (Table 2).

**Table 2:** Patient’s characteristics at baseline

Study, year	*n*° patients	Age, mean	Female, *n*° (%)	*n*° previous line of treatment, mean	Previous ASCT, %	EMD, %	High cytogenetic risk, %	ISS III, %	ISS I, %	BCMA exposed, %	Penta-refractory, %
Mohan, 2024	110	67.5	54 (49)	6,2	87	44	62*	n.a.	n.a.	35	76
Riedhammer, 2024	123	66.4	53 (43.1)	6.2	n.a.	36.1*	36.8*	33.7	27.1	37.4	60.2
Mian fit, 2025	22	73.1	9 (40.9)	7.2	n.a.	n.a.	61.1	27.3	27.3	50	40
Mian frail, 2025	59	78.4	35 (40.7)	6	n.a.	n.a.	55.3	33.3	28.1	33.9	48.2
Razzo, 2025	509	67.8	234 (46)	6.1	65	45*	54	n.a.	n.a.	46.4	38
Stork nwd, 2025	18	68.1	10 (55.6)	4.6	n.a.	11.1	28.6*	33.3	27.8	5.6	55.6
Stork wd, 2025	55	65.5	27 (49.1)	5.5	n.a.	29.1	48.9*	23.6	30.9	14.5	88.9
Tan, 2025	210	66.7	93 (44.3)	6.3	n.a.	29.4*	50*	30.6	37.9	43.8	44.1
Yi, 2025	42	66.8	15 (35.7)	6.1	55’	28.4	41.5	n.a.	n.a.	7.1	19
Hammons comb, 2023	15	64.1	5 (33)	6	93	n.a.	n.a.	n.a.	n.a.	n.a.	n.a.
Hammons mono, 2023	15	69.8	7 (47)	6.3	93	n.a.	n.a.	n.a.	n.a.	n.a.	n.a.
Frenking, 2024	138	63.1	42 (30)	6.2	86	48*	48*	43	30	51	47
Cani, 2025	57	64.8	n.a.	6.6	95	n.a.	n.a.	n.a.	n.a.	75	n.a.
Total	**1**,**373**	**67.9**	**584 (42.9)** ^§^	**6.1**	**82** ^§^	**34** ^§^	**48** ^§^	**29.7** ^§^	**29.9** ^§^	**36.3** ^§^	**51.7** ^§^

*ASCT*, autologous stem cell transplant; *BCMA*, B-Cell maturation antigen; *EMD*, extramedullary disease; *ISS*, international staging system; *n.a.*, not available

*Percentage is calculated based on total of patients assessed for that clinical manifestation

^§^The denominator does not include patients that were not assessed for that clinical manifestation

### Severe infection rate

**Fig. 2:**
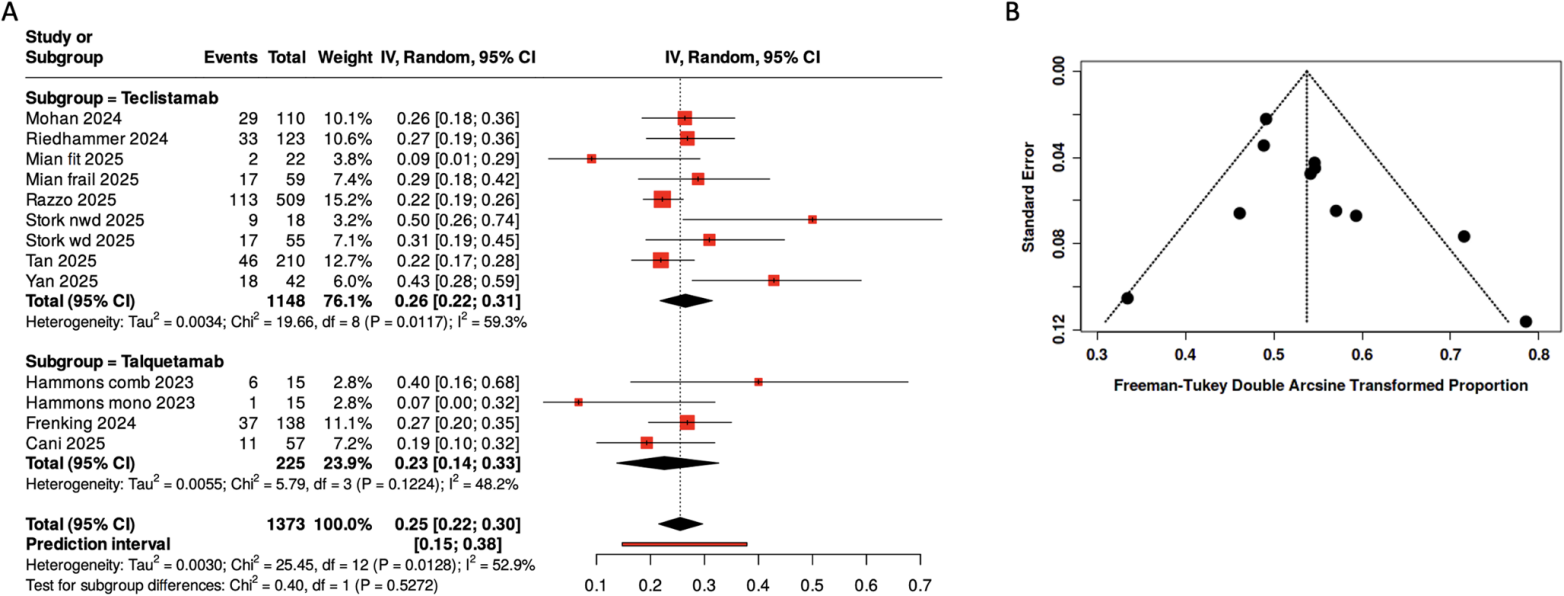
Meta-analysis of grade 3–4 infections in the included studies, funnel plot and meta-regression analysis. **A** Meta-analysis of patients with multiple myeloma assessing grade 3–4 infection events. Effects are expressed as pooled event rates with 95% confidence intervals (CIs). **B** Funnel plot under random effect model. Egger’s test did not indicate statistically significant asymmetry (intercept = 1.0, 95% CI: − 0.57 to 2.58, *p* = 0.237)

A total of 339 patients (24.7%) experienced grade 3–4 infections after a weighted median follow-up of 8.3 months, corresponding to a pooled event rate of 0.25 (95% CI, 0.22–0.30) as shown in Fig. 2A. Moderate heterogeneity was observed (Tau²=0.0030; Chi²=25.45, df = 12, *p* = 0.0128; *I*²=52.9%). In Fig. 2B, Egger’s test did not indicate significant funnel plot asymmetry (intercept = 1.0, 95% CI, -0.57 to 2.58; *p* = 0.237). In the subgroup analysis, studies evaluating teclistamab included a total of 1,148 patients, with a pooled event rate of 0.26 (95% CI, 0.22–0.31; *I*²=59.3%). Studies assessing talquetamab comprised 225 patients, showing a pooled event rate of 0.23 (95% CI, 0.14–0.33) with moderate heterogeneity (*I*²=48.2%).

**Fig. 3:**
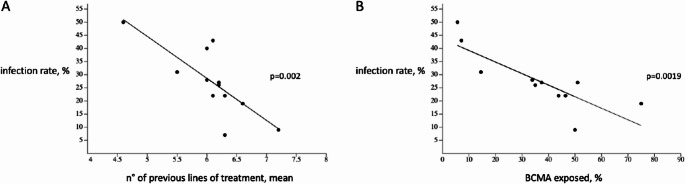
Meta-regression analysis for n° of previous line of treatment and percentage of BCMA exposed patients prior BiTES therapy. **A** Meta-regression analysis for n° of previous lines of treatment (mean) for each study on infection rate (%). **B** Meta-regression analysis for percentage of BCMA exposed patients for each study on infection rate (%)

Given the observed heterogeneity, meta-regression identified a significant inverse association between severe infection rates and both prior lines of therapy (*p* = 0.002) and prior BCMA exposure (*p* = 0.0019), as shown in Fig. 3. Studies including more heavily pretreated or BCMA-exposed patients reported lower infection rates. Indeed, when outlier studies were excluded from the pooled analysis, heterogeneity markedly decreased to 7.5%, with a pooled event rate of 0.24 (95% CI, 0.21–0.26), as shown in S-Figure 1. Interestingly, when considering the two treatment subgroups, the pooled event rate for teclistamab and talquetamab overlapped: 0.24 (95% CI, 0.21–0.27) and 0.24 (95% CI, 0.18–0.31), respectively.

### Other BiTEs-related adverse events

Across the included studies, the overall incidence of cytokine release syndrome (CRS) of any grade was 55.1%, while immune effector cell-associated neurotoxicity syndrome (ICANS) occurred in 9% of patients. The pooled overall response rate (ORR) was 63%, and immunoglobulin supplementation was reported in 56% of cases. Unfortunately, not all studies reported data for every variable (S-Table 3).

## Discussion

BiTEs have significantly improved outcomes for patients with RRMM, but their expanding real-world use makes infectious safety a key concern. In MM, infection risk is multifactorial, driven by disease-related immune dysfunction, prior therapies, and profound hypogammaglobulinemia induced by T-cell–redirecting agents. Defining severe infection incidence and patterns is essential to optimize supportive care and treatment continuity.

This meta-analysis synthesizes real-world data on grade 3–4 infections in MM patients treated with teclistamab or talquetamab, two of the BiTEs currently approved and those supported by the most mature real-world evidence for clinical use in RRMM. Across 10 retrospective cohorts including 1,373 patients, with a median follow-up of 8.3 months, the pooled event rate was 25%, consistent with the pivotal trials previously reported. Notably, the imbalance between teclistamab and talquetamab studies (7 vs. 3) reflects the current landscape of real-world evidence, which is still evolving and remains more extensive for these agents due to longer clinical experience. Moderate heterogeneity was observed, and no evidence of publication bias was detected. In the subgroup analysis, the pooled event rate for teclistamab was 0.26 (95% CI, 0.22–0.31), while for talquetamab it was 0.23 (95% CI, 0.14–0.33), with comparable heterogeneity, suggesting that severe infections may represent a class-related toxicity of T-cell-redirecting therapies rather than a strictly target-specific effect.

Compared with pivotal trials, our studies estimate lies within the lower reported range. In MajesTEC-1, grade 3–4 infections with teclistamab reached 44.8% at 14 months, while in MonumenTAL-1, talquetamab was associated with 18–26% severe infections [4, 6]. While BCMA-directed BiTEs appeared to confer a numerically higher infectious risk in pivotal trials, this difference was not observed in our real-world analysis. Shorter follow-up, clinician-driven patient selection, and prophylactic measures after early BCMA safety signals may have attenuated between-agent differences, and the limited talquetamab sample limits statistical power.

Meta-regression revealed a paradoxical inverse association between infection rates and both number of prior treatment lines and prior BCMA exposure (*p* = 0.002 and *p* = 0.0019, respectively), as shown in Fig. 3. This likely reflects real-world selection bias: clinicians tend to offer BiTEs to fitter, immunologically stable patients, so more heavily pretreated cohorts may represent resilient individuals. This interpretation is supported by the higher infection rate reported in MajesTEC-1 (about 45%) compared with our pooled analysis, whereas talquetamab rates (24%) were broadly consistent with MonumenTAL-1 (≤ 26%). Notably, only three retrospective studies assessed talquetamab, limiting precision. Biological variability in immune competence and tumor-immune microenvironment may also influence both efficacy and infection susceptibility [27].

Previous meta-analyses reported similar findings. Wang et al. [28] found grade ≥ 3 infections in about 30% of patients receiving BiTEs, including both BCMA-targeting agents (e.g., teclistamab, elranatamab, linvoseltamab) and non-BCMA-targeting agents (e.g., talquetamab and cevostamab). Vandenboom et al. [29] likewise reported a 30% pooled rate with BCMA-targeting BiTEs (e.g., teclistamab and elranatamab). Together, these data reinforce infections as a consistent class effect of T-cell-redirecting therapies.

Limitations must be considered. The included studies were predominantly retrospective real-world analyses, subject to selection bias, incomplete data capture, and reporting variability, thus providing lower-quality evidence than randomized trials. Differences in patient populations, follow-up, and outcome definitions further limit cross-trial comparability. These findings should therefore be considered hypothesis-generating, particularly regarding the apparent similarity in grade 3–4 infection rates. Temporal heterogeneity in infection prevention and supportive care may also have influenced results. Inconsistent reporting of infection prophylaxis limited adjustment for its impact, and the imbalance in studies on teclistamab and talquetamab further restricts comparability.

Standardized tools to assess infection risk - integrated with validated instruments evaluating patient frailty - and prospective registries with harmonized pharmacovigilance are needed to refine prevention strategies and ensure the safe integration of BiTEs into MM care.

In conclusion, this study provides the first real-world estimate of severe infections in RRMM patients treated with approved BiTEs (teclistamab and talquetamab). About 25% developed grade 3–4 infections over 8.3 months of follow-up, confirming that infection remains a major clinical challenge and supporting systematic monitoring and preventive strategies in routine practice.

## Supplementary Information

Below is the link to the electronic supplementary material.

Supplementary Material 1

## Data Availability

Data will be provided by the author upon reasonable request.
