# Supplementary material for: Real-world incidence of severe infections in multiple myeloma patients receiving bispecific antibodies: a meta-analysis
Source: Ann Hematol. 2026 Apr 27;105(5):269. doi: 10.1007/s00277-026-07026-9 (PMC13121584; doi:10.1007/s00277-026-07026-9)
Supplement: Supplementary file 1 — Supplementary Material 1 [file 277_2026_7026_MOESM1_ESM.docx]

S-Table 1. Quality assessment of the studies.

| Study | **1** | **2** | **3** | **4** | **5** | **6** | **7** | **8** | **9** | **10** | **11** | **12** | **13** | **14** | **15** | **16** | **17** | **18** | **19** | **20** | **Quality score** |
| --- | --- | --- | --- | --- | --- | --- | --- | --- | --- | --- | --- | --- | --- | --- | --- | --- | --- | --- | --- | --- | --- |
| ***Teclistamab*** |  |  |  |  |  |  |  |  |  |  |  |  |  |  |  |  |  |  |  |  |  |
| *Mohan*  *2024* | Y | N | Y | Y | Y | Y | Y | Y | Y | Y | N | Y | Y | Y | Y | Y | Y | Y | Y | Y | 18/20 |
| *Riedhammer*  *2024* | Y | N | Y | Y | Y | Y | Y | Y | Y | Y | N | Y | Y | Y | Y | Y | Y | Y | Y | Y | 18/20 |
| *Mian*  *2025* | Y | N | Y | Y | Y | Y | Y | Y | Y | Y | N | Y | Y | Y | Y | Y | Y | Y | Y | Y | 18/20 |
| *Razzo*  *2025* | Y | N | Y | Y | Y | Y | Y | Y | Y | Y | N | Y | Y | Y | Y | Y | Y | Y | Y | Y | 18/20 |
| *Stork*  *2025* | Y | N | Y | Y | Y | Y | Y | Y | Y | Y | N | Y | Y | Y | Y | Y | Y | Y | Y | Y | 18/20 |
| *Tan*  *2025* | Y | N | Y | Y | Y | Y | Y | Y | Y | Y | N | Y | Y | Y | Y | Y | Y | Y | Y | Y | 18/20 |
| *Yi*  *2025* | Y | N | Y | Y | Y | Y | Y | Y | Y | Y | N | Y | Y | Y | Y | Y | Y | Y | Y | Y | 18/20 |
| ***Talquetamab*** |  |  |  |  |  |  |  |  |  |  |  |  |  |  |  |  |  |  |  |  |  |
| *Hammons*  *2023* | Y | N | Y | Y | Y | Y | Y | Y | Y | Y | N | Y | Y | Y | Y | Y | Y | Y | Y | Y | 18/20 |
| *Frenking*  *2024* | Y | N | Y | Y | Y | Y | Y | Y | Y | Y | N | Y | Y | Y | Y | Y | Y | Y | Y | Y | 18/20 |
| *Cani*  *2025* | Y | N | N | Y | Y | Y | Y | Y | Y | Y | N | Y | Y | Y | Y | Y | Y | Y | Y | Y | 17/20 |

Y = YES; N = NO; P = PARTIAL; U = UNCLEAR. A study with ≥ 70% of positive responses (12/18 or 14/20) was considered to be of acceptable quality.

Institute of Health Economics (IHE). Quality Appraisal of Case Series Studies Checklist. Edmonton (AB): Institute of Health Economics; 2014. Available from: <http://www.ihe.ca/research-programs/rmd/cssqac/cssqac-about>

References:

Moga C, Guo B, Schopflocher D, Harstall C. Development of a quality appraisal tool for case series studies using a modified Delphi technique. Edmonton: Institute of Health Economics; 2012. Available at <http://www.ihe.ca/advanced-search/development-of-a-quality-appraisal-tool-for-case-series-studies-> [using-a-modified-delphi-technique](http://www.ihe.ca/advanced-search/development-of-a-quality-appraisal-tool-for-case-series-studies-using-a-modified-delphi-technique) . Accessed January 8, 2016.

Guo B, Moga C, Harstall C, Schopflocher D. A principal component analysis is conducted for case series quality appraisal checklist. Journal of Clinical Epidemiology 2016;69:199-207.

S-Table 2. Summary of Findings Table.

| **Certainty assessment** | | | | | | | **№ of patients** | | **Effect** | | **Certainty** | **Importance** |
| --- | --- | --- | --- | --- | --- | --- | --- | --- | --- | --- | --- | --- |
| **№ of studies** | **Study design** | **Risk of bias** | **Inconsistency** | **Indirectness** | **Imprecision** | **Other considerations** | **Treated** | **Untreated** | **Relative (95% CI)** | **Absolute (95% CI)** |  |  |
| **Infection risk (follow-up: mean 45 weeks; assessed with: mg)** | | | | | | | | | | | | |
| 10^a^ | non-randomised studies | serious^b^ | not serious^c^ | not serious | not serious^d^ | publication bias suspected^e^ | 1,373 | -^f^ | - | **0.25**  (0.22 to  0.30) | ⨁⨁◯◯  Low | CRITICAL |

**CI:** confidence interval.

**Explanations**

a. All included studies scored above 70% on the IHE checklist, indicating good methodological quality.

b. All included studies were retrospective observational analyses. As such, they are subject to inherent limitations, including potential selection bias, incomplete outcome reporting, and variability in follow-up and infection ascertainment. However, the outcome (grade 3–4 infections) is relatively objective, which mitigates some risk. Overall, the risk of bias was considered serious**.**

c. Moderate heterogeneity was observed (I²=52.9%, p=0.0128), indicating some variability among study estimates. However, the direction of the effect was consistent across studies, and the confidence interval of the pooled proportion was relatively narrow (0.22–0.30). Therefore, inconsistency was judged not serious.

d. The pooled infection rate was 0.25 (95% CI 0.22-0.30), with a narrow confidence interval indicating a precise estimate of the proportion of patients experiencing infection. The sample size and number of events were sufficient to ensure statistical stability, and therefore imprecision is not considered serious.

e. Publication bias was assessed using a funnel plot of Freeman–Tukey transformed proportions and further tested with Egger’s regression. The funnel plot showed mild asymmetry, with two studies lying outside the 95% confidence limits, suggesting potential small-study effects or selective reporting. However, Egger’s test did not indicate statistically significant asymmetry (intercept=1.0, 95% CI: –0.57 to 2.58, p=0.237). Given the limited number of included studies and the known challenges in interpreting funnel plots for proportion-based meta-analyses, the certainty of the evidence was downgraded by one level for suspected publication bias.

f. Control group are not considered in this metanalysis.

S-Table 3. Severe infection rate, BiTES-related toxicities, overall remission rate and substitutive immunoglobulins use across the studies.

| **study,**  ***year*** | **n° patients** | **patients with Grade 3-4 infection,**  ***n°* (%)** | **CRS all grade, %** | **ICANS, %** | **ORR, %** | **Immunoglobulins supplementation, %** |
| --- | --- | --- | --- | --- | --- | --- |
| Mohan, 2024 | 110 | 29 (26) | 56 | 11 | 62 | 43 |
| Riedhammer, 2024 | 123 | 33 (26.8) | 58.5 | 7.3 | 59.3 | n.a. |
| Mian fit, 2025 | 22 | 2 (9.1) | 54.6 | 9.1 | 55 | n.a. |
| Mian frail, 2025 | 59 | 17 (28.8) | 47.5 | 13.6 | 66.1 | n.a. |
| Razzo, 2025 | 509 | 113 (22) | 54 | 11 | 61 | 38 |
| Stork nwd, 2025 | 18 | 9 (50) | 44.4 | 11.1 | n.a. | 72.2 |
| Stork wd, 2025 | 55 | 17 (31) | 49.1 | 0 | n.a. | 77.4 |
| Tan, 2025 | 210 | 46 (21.9) | 54 | 7.3 | 67 | 62 |
| Yi, 2025 | 42 | 18 (42.9) | 47.6 | 0 | 66.7 | 50 |
| Hammons comb, 2023 | 15 | 6 (40) | 93 | 13 | n.a. | 73 |
| Hammons mono, 2023 | 15 | 1 (6.7) | 67 | 13 | n.a. | 20 |
| Frenking, 2024 | 138 | 37 (26.8) | 70 | 9 | 65 | 50 |
| Cani, 2025 | 57 | 11 (19) | 21 | 11 | n.a. | 72 |
| **total** | **1,373** | **339 (24.7)** | **55.1** | **9** | **63**^§^ | **56**^§^ |

CRS, cytokine release syndrome; ICANS, immune effector cell-associated neurotoxicity syndrome; n.a., not available; ORR, overall response rate.

^§^The denominator does not include patients that were not assessed for that clinical manifestation.

S-Figure 1. Meta-analysis excluding outlier studies both for teclistamab and talquetamab treated patients


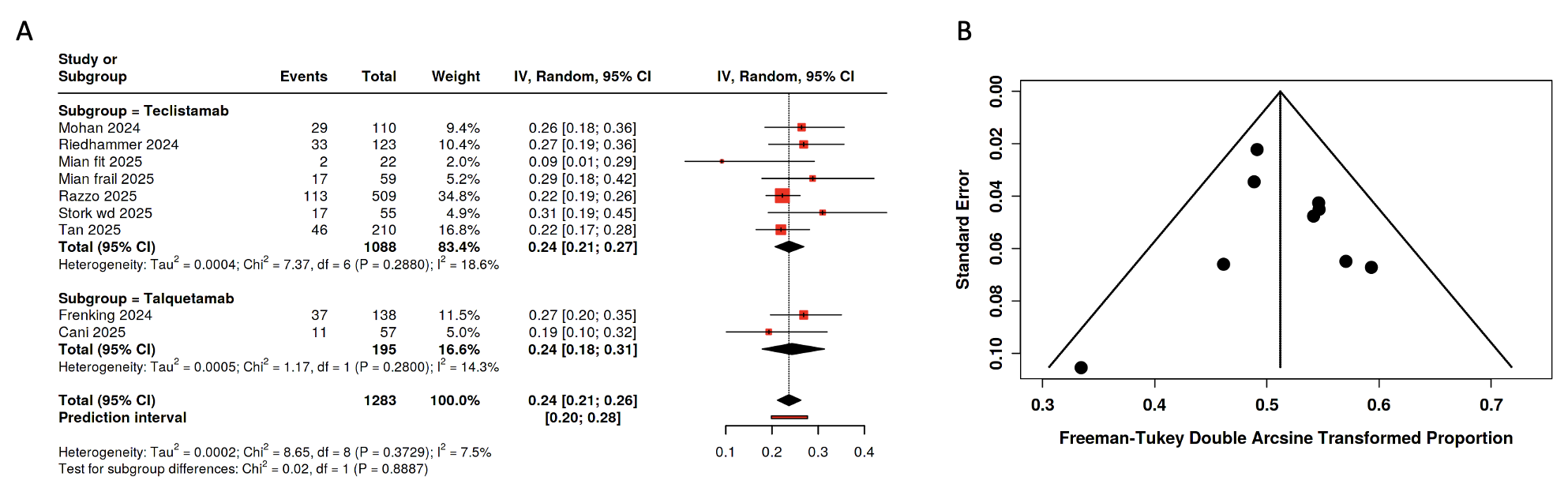


Meta-analysis of patients with multiple myeloma assessing grade 3-4 infection events excluding outlier studies reaching low I^2^. Effects are expressed as pooled event rates with 95% confidence intervals (CIs). B) Based on the analysis performed using a random effects model with the inverse variance method and Freeman-Tukey Double arcsine transformation, the summarized proportion is 0.24 with a 95% CI of 0.22 - 0.27.
